# Transcriptional Fingerprint of Hypomyelination in *Zfp191*^*null*^ and *Shiverer* (*Mbp*^*shi*^) Mice

**DOI:** 10.1177/1759091416670749

**Published:** 2016-09-28

**Authors:** Joshua D. Aaker, Benayahu Elbaz, Yuwen Wu, Timothy J. Looney, Li Zhang, Bruce T. Lahn, Brian Popko

**Affiliations:** 1 Department of Neurology, The University of Chicago Center for Peripheral Neuropathy, The University of Chicago, IL, USA; 2 Department of Human Genetics, The University of Chicago, IL, USA

**Keywords:** cholesterol biosynthesis, hypomyelination, oligodendrocyte development, shiverer, transcriptional networks, ZFP191

## Abstract

The transcriptional program that controls oligodendrocyte maturation and central nervous system (CNS) myelination has not been fully characterized. In this study, we use high-throughput RNA sequencing to analyze how the loss of a key transcription factor, zinc finger protein 191 (ZFP191), results in oligodendrocyte development abnormalities and CNS hypomyelination. Using a previously described mutant mouse that is deficient in ZFP191 protein expression (*Zfp191*^*null*^), we demonstrate that key transcripts are reduced in the whole brain as well as within oligodendrocyte lineage cells cultured *in vitro*. To determine whether the loss of myelin seen in *Zfp191*^*null*^ mice contributes indirectly to these perturbations, we also examined the transcriptome of a well-characterized mouse model of hypomyelination, in which the myelin structural protein myelin basic protein (MBP) is deficient. Interestingly, *Mbp*^*shi*^ (shiverer) mice had far fewer transcripts perturbed with the loss of myelin alone. This study demonstrates that the loss of ZFP191 disrupts expression of genes involved in oligodendrocyte maturation and myelination, largely independent from the loss of myelin. Nevertheless, hypomyelination in both mouse mutants results in the perturbation of lipid synthesis pathways, suggesting that oligodendrocytes have a feedback system that allows them to regulate myelin lipid synthesis depending on their myelinating state. The data presented are of potential clinical relevance as the human orthologs of the *Zfp191* and *MBP* genes reside on a region of Chromosome 18 that is deleted in childhood leukodystrophies.

## Introduction

Oligodendrocytes are the cells that produce myelin in the vertebrate central nervous system (CNS). The majority of oligodendrocyte progenitor cells (OPCs) are derived from neural progenitor cells in the subventricular zone (Richardson et al., 2006). During CNS development, OPCs migrate along vasculature to their target axons where they terminally differentiate to mature oligodendrocytes, express myelin genes, produce myelin, wrap axons, and provide metabolic axonal support (Simons and Nave, 2015; Tsai et al., 2016).

OPCs are able to proliferate until they transit to their target axons where they undergo terminal differentiation, which is achieved through a delicate balance of both inhibitors and promoters of differentiation (Menn et al., 2006). Among the inhibitors of oligodendrocyte differentiation, the G-protein-coupled receptor 17 is an oligodendrocyte-specific receptor that strongly inhibits differentiation and may work by increasing the expression of inhibitors of differentiation 2 and 4 (ID2/ID4; Wang et al., 2001; Chen et al., 2009). ID2/ID4 along with Hes family bHLH transcription factors 1 and 5 (HES1/5) have been shown to enforce the proliferating OPC state and to repress terminal differentiation (Wu et al., 2003; Liu et al., 2006). In addition, the Notch and Wnt signaling pathways have been implicated in suppressing oligodendrocyte differentiation (Wang et al., 1998; Fancy et al., 2009; Nakatani et al., 2013). Wnt signals were shown to be upstream of bone morphogenetic protein signaling (Feigenson et al., 2011), which was shown to inhibit oligodendrocyte differentiation (Grinspan et al., 2000; Miller et al., 2004; Samanta and Kessler, 2004; See et al., 2004; Cheng et al., 2007). Despite the inhibitory effect of the Wnt signaling pathway on oligodendrocyte differentiation, the Wnt effector transcription factor 4 promotes oligodendrocyte differentiation (Fancy et al., 2009; Hammond et al., 2015).

Among the promoters of differentiation, oligodendrocyte transcription factor 2 promotes differentiation of oligodendrocytes by recruiting the chromatin-remodeling enzyme SWI/SNF related, matrix associated, actin-dependent regulator of chromatin, subfamily a, member 4 (SMARCA4/BRG1) to regulatory elements of key genes, including *Zfp191* and *Sox10* during differentiation (Yu et al., 2013). SRY (sex determining region Y)-box 10 (SOX10) is required for the generation of myelinating oligodendrocytes and is a direct activator of several myelin-related genes (Li et al., 2007). One of the genes controlled by SOX10 is myelin regulatory factor (*MYRF*; Hornig et al., 2013). SOX10 binds in the first intron of the *MYRF* gene and induces its expression during oligodendrocyte differentiation. Following its induction, *MYRF* mediates the progression of premyelinating oligodendrocytes to a mature, myelinating state (Emery et al., 2009). *MYRF* and SOX10 target many of the same myelin gene enhancers and promoters but also appear to target individual enhancers independently of the other (Bujalka et al., 2013; Hornig et al., 2013).

Previous work from our laboratory (Howng et al., 2010) has demonstrated that zinc finger protein 191 (ZFP191, also known as ZFP24) is required for oligodendrocyte differentiation. We have shown that many of the oligodendrocyte-specific genes induced during oligodendrocyte differentiation (including abundantly expressed myelin genes such as *Mbp* and *Plp1*) are reliant on ZFP191 for their expression (Howng et al., 2010); however, the full spectrum of genes that are affected by loss of ZFP191 was unknown. Therefore, we characterize here the effect of *Zfp191* loss on the whole transcriptome by high-throughput RNA sequencing (RNA-seq).

ZFP191 contains a SCAN domain that may be involved in protein–protein interaction (Williams et al., 1999) and four C_2_H_2_ zinc finger domains that bind DNA (Wang et al., 2008). ZFP191 is a member of the C_2_H_2_ zinc-finger protein family, many members of which are known to be DNA binding proteins that function as transcriptional regulators (Edelstein and Collins, 2005). Consistent with this, we find that loss of *Zfp191* results in substantial changes in the transcriptome of the whole brain and in the transcriptome of cultured oligodendrocyte lineage cells.

To examine whether the substantial changes in the transcriptome in the *Zfp191*^null^ mice CNS are due to the direct loss of ZFP191 or whether they represent a secondary consequence of the inability to produce myelin, we performed RNA sequencing using the *shiverer* mouse, which is a model of hypomyelination that resulted from a spontaneous null mutation in the gene encoding the structural myelin protein, myelin basic protein (MBP; Roach et al., 1985). We find that unlike hypomyelination originating from the lack of *Zfp191*, the effect of the *MBP* mutation on the transcriptome was minor, suggesting that the loss of ZFP191 disrupts transcripts involved in oligodendrocyte maturation and myelination largely independent from the loss of myelin. Interestingly, the loss of myelin in both mouse mutants, however, results in the perturbation of the cholesterol biosynthesis pathway.

## Materials and Methods

### Animal Work

Generation of *Zfp191*^*null*^ mice on the C57BL/6J background has been previously described (Howng et al., 2010). The C3Fe.SWV-*Mbp*^*shi*^/J *shiverer* strain of mice was purchased from The Jackson Laboratory (stock 001428) and has been previously described (Readhead et al., 1987). All animal procedures were conducted in complete compliance with the National Institutes of Health Guide for the Care and Use of Laboratory Animals and were approved by the Institutional Animal Care and Use Committee of the University of Chicago.

### Cell Culture

Primary OPCs were isolated from brain and enzymatically and mechanically dissociated as previously described (Emery and Dugas, 2013). Briefly, both cortices were removed and cells were dissociated by enzymatic and mechanical methods. The single cell suspension was then immunopanned on two plates coated with 50 mM Tris-HCl pH = 9.5 and goat anti-mouse IgG + IgM (Jackson ImmunoResearch, 115-055-044) and one plate coated in 50 mM Tris-HCl with goat anti-mouse IgM, µ-chain specific (Jackson ImmunoResearch, 115-005-020). These plates were then sequentially incubated with rat neural antigen 2 (Ran-2), galactocerebroside (GC), and oligodendrocyte marker (O4) hybridomas, respectively, in 0.2% bovine serum albumin and Dulbecco's phosphate-buffered saline (Life Technologies #14040133). The plates served as two sequential negative selection plates coated with Ran-2 and GC hybridomas followed by a positive selection plate coated with O4 hybridoma. The Ran-2 immunopanning captures type 1 astrocytes and meningeal cells (Bartlett et al., 1981). The GC immunopanning captures differentiated oligodendrocytes (Sommer and Schachner, 1981). The O4 hybridoma is able to capture O-2A progenitor cells (Sommer and Schachner, 1981). Cells were then trypsinized (0.25% in Earle's balanced salt solution, Life Technologies #14155063) and plated on poly-d-lysine coated plates. Cells were maintained in Sato serum-free Dulbecco's modified Eagle medium (Life Technologies #11960069) as previously described with the addition of B27 supplement (Life Technologies #17504044; Dugas et al., 2006). For proliferation media, platelet-derived growth factor-AA (PDGF-AA; 10 ng/ml, PeproTech #100-13A), neurotrophin-3 (1 ng/ml, PeproTech #450-03), forskolin (Sigma Aldrich [0.01 mM]), and ciliary neurotrophic factor (CNTF; 10 ng/ml, PeproTech #450-13) were added. To stimulate differentiation, PDGF-AA was removed and triiodothyronine (40 ng/ml, Sigma # T6397) was added. Media was changed every other day and differentiated cells were collected after 5 days.

### RNA Sequencing

For whole-brain RNA sequencing, mice were anesthetized with avertin (0.5% 2,2,2-tribromoethanol; Sigma-Aldrich #T48402; w/v), 0.5% tert-amyl alcohol; Fluka #PHR1667; v/v) in MillQ water) used at 100 μl per 10 g body weight, and the cerebral hemispheres were removed and snap frozen at −80 ℃ at postnatal Day 21. Total RNA was isolated using Aurum Total RNA Fatty and Fibrous Tissue Kit (Bio-Rad #732-6830) following the manufacturer's protocol. OPC and oligodendrocyte total RNA was collected using Aurum Total RNA Kit (Bio-Rad #732-6820). Two biological replicates were used for all samples. The quality of the RNA was assessed using an Agilent 2100 Bioanalyzer. RNA sequencing libraries were prepared using Illumina TruSeq RNA sample Kit (#RS-122-2001) and sequenced by HiSeq 2000 and HiSeq 2500 sequencers using HiSeq SBS Kit (Illumina #FC-401-4002) at the University of Chicago Functional Genomics Facility. Sequencing data have been uploaded to Sequence Read Archive from the National Center for Biotechnology Information through the National Library of Medicine under accession number PRJNA338665.

### Bioinformatics

RNA sequencing reads were mapped to the mm9 genome using BowTie v.1 and the ExpressionPlot pipeline (Langmead et al., 2009; Friedman and Maniatis, 2011). Differential expression was performed using DEseq in the ExpressionPlot software package (Anders and Huber, 2010). The Bonferroni correction was used within DEseq to control for multiple comparisons. Only transcripts ≥0.5 Reads per Kilobase per Million (RPKM) mapped reads, ≥1.5-fold change, and *p* < .001 were analyzed. *p* Value of 8.28 × 10^−307^ or lower was rounded to zero. Kyoto Encyclopedia of Genes and Genomes (KEGG) pathway analysis was performed using DAVID (Kanehisa and Goto, 2000; Huang da et al., 2009a, 2009b; Kanehisa et al., 2014). The Galaxy environment was used to compare datasets (Giardine et al., 2005; Blankenberg et al., 2010; Goecks et al., 2010). Cellular identity stratification was done using the online RNA-Seq transcriptome and splicing database (http://web.stanford.edu/group/barres_lab/brain_rnaseq.html; Zhang et al., 2014). The top 500 transcripts for each cell identity from the database were compiled in order to allow for cell-type comparison and grouping from our results.

### Real-Time PCR

RNA was collected using Aurum Total RNA Kit (Bio-Rad #732-6820). RNA integrity was verified using Agilent chip (Agilent Technologies). cDNA was generated using iScript cDNA Synthesis Kit (Bio-Rad **#**1708890) following the manufacturer's protocol. Real-time polymerase chain reaction (PCR) was performed on the cDNA using iQ SYBR Green Supermix (Bio-Rad #1708882) using a CFX96 Touch Deep Well Real-Time PCR Detection System (Bio-Rad #1854095). Each sample was done in triplicate. Relative expression from amplified cDNA samples was determined using the ${2^{-\Delta \Delta }}_{\text{CT}}$ method (Pfaffl, 2001). The primers sequences for all the transcripts that were selected for analysis are in Table S1. The expression of the selected transcripts was normalized to the housekeeping gene hypoxanthine-guanine phosphoribosyltransferase (*Hprt*) cDNA.

## Results

### Loss of *Zfp191* Expression Perturbs the Whole Brain Transcriptome

Previous work has shown that ZFP191 is required for CNS myelination. Nonetheless, the role that ZFP191 plays in normal myelin production by oligodendrocytes is not well understood (Howng et al., 2010). To examine the potential role of ZFP191 in transcriptional regulation of CNS myelination, we used RNA sequencing to examine changes in the transcriptome. RNA sequencing was performed on postnatal Day 21 (P21) and whole-brain RNA samples were taken from *Zfp191*^*null*^ and *Zfp191*^*+/+*^ littermates. The levels of 394 transcripts were determined to be significantly different in the *Zfp191*^*null*^ brains compared with *Zfp191*^*+/+*^ brains (Tables S2 and S3). The majority of the perturbed *Zfp191*^*null*^ brain transcripts displayed reduced levels of expression in the mutant animals.

The loss of ZFP191 resulted in the decreased expression of 327 transcripts when compared with the *Zfp191*^*+/+*^ CNS transcriptome (Table S2). Several transcripts that encode key myelin proteins, such as MBP and myelin oligodendrocyte glycoprotein (MOG; Matthieu and Amiguet, 1990), were identified as having decreased transcript levels. We also observed that transcripts that encode key oligodendrocyte transcriptional factors such as SOX10 and MYRF had reduced expression with loss of ZFP191 along with downstream targets of MYRF, such as the transcript for ring finger and FYVE-like domain containing E3 ubiquitin protein ligase (*Rffl*; Bujalka et al., 2013).

Table 1A and B show the top 10 transcripts based on expression level, as determined by RPKM values, and fold change, respectively, that have reduced expression in the *Zfp191*^*null*^ whole brain. Transcripts with reduced expression in the *Zfp191*^*null*^ brain were analyzed for changes in characterized pathways using the KEGG (Kanehisa and Goto, 2000; Kanehisa et al., 2014). The steroid biosynthesis pathway is significantly disturbed due to disruption of transcripts that encode key biosynthetic pathway proteins such as 3-hydroxy-3-methylglutaryl-CoA reductase (HMGCR), fatty acid 2-hydroxylase (FA2H), and 24-dehydrocholesterol reductase (Saher and Simons, 2010; Table S2). Proper synthesis of cholesterol is known to be required for production of compact myelin in oligodendrocytes (Saher et al., 2005).

**Table 1.** Top 10 Transcripts That Have Reduced Expression in *Zfp191*^*null*^ Whole Brains.

Gene	Fold change	*Zfp191*^*+/+*^ RPKM
A. Genes sorted by *Zfp191*^*+/+*^ transcript RPKM		
*Plp1*	8.39	1210.00
*Mbp*	6.06	749.00
*Cldn11*	6.62	258.00
*Mal*	9.57	222.00
*Cnp*	3.33	207.00
*Mobp*	13.82	129.00
*Mag*	10.87	117.00
*Mog*	7.27	111.00
*Ugt8a*	10.56	111.00
*Tspan2*	5.20	110.00
B. Genes sorted by fold change for *Zfp191*^*+/+*^		
*Gjb1*	15.08	14.90
*Mobp*	13.82	129.00
*Gjc2*	11.53	12.20
*Mag*	10.87	117.00
*Plekhh1*	10.63	7.72
*Ugt8a*	10.56	111.00
*A230069A22Rik*	9.73	24.90
*Mal*	9.58	222.00
*Ermn*	9.12	35.10
*Plp1*	8.39	1210.00

RNA sequencing was performed on *Zfp191*^*null*^ and *Zfp191*^*+/+*^ whole-brain RNA samples (*n* = 2 for each genotype). Transcripts listed for *Zfp191*^*+/+*^ whole brains in A had a *p* < .001 fold change when comparing *Zfp191*^*+/+*^ versus *Zfp191*^*null*^ whole brains and are sorted by RPKM value. Transcripts listed for *Zfp191*^*+/+*^ whole brains in B had a RPKM value ≥7, a *p* < .001 fold change when comparing *Zfp191*^*+/+*^ versus *Zfp191*^*null*^ whole brains, and are sorted by fold changes.

### Loss of *Zfp191* Expression Mainly Affects Cell-Specific Transcripts in the Oligodendrocyte Lineage Along With Key Signaling Cascades

**Figure 1.**
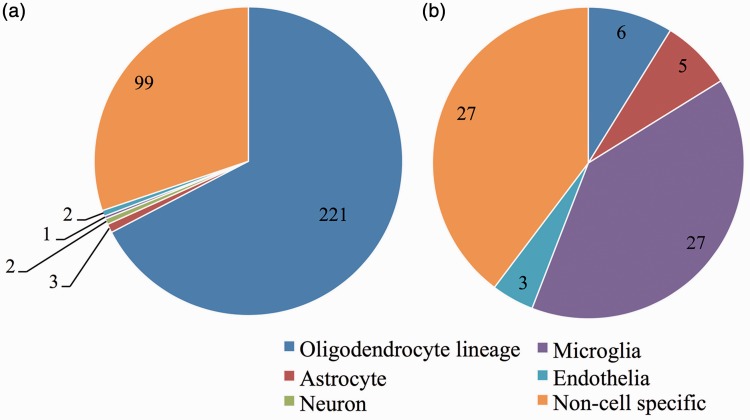
Loss of *Zfp191* expression mainly perturbs transcripts associated with the oligodendrocyte lineage in the whole brain. Number of transcripts with decreased expression by loss of ZFP191 (A) or increased expression (B) sorted into cellular identity categories using data from Zhang et al. (Zhang et al., 2014). *Note*. ZFP191 = zinc finger protein 191.

To determine the cells that are most affected by the loss of ZFP191 in the brain, we stratified the 394 differentially expressed transcripts (Tables S2 and S3) into cell-specific identities (oligodendrocyte lineage, neuron, astrocyte, microglia, endothelia, or noncell specific) using the online RNA-Seq transcriptome and splicing database (Zhang et al., 2014). Strikingly, of the 327 transcripts that had lower expression levels with loss of ZFP191, 221 transcripts were associated with oligodendrocyte lineage cells (Figure 1).

Loss of *Zfp191* expression increased the mRNA levels of 67 transcripts in the mutant CNS; 10 transcripts with the most abundant RPKM are listed in Table 2 (the full list is in Table S3). One transcript that was increased encodes glial fibrillary acidic protein (*Gfap*; Bignami et al., 1972), which may suggest astrogliosis. This is supported by a 2.5-fold increase in serine peptidase inhibitor, clade A, member 3N (*Serpina3n)*, which has been shown to display increased expression in reactive astrocytes (Zamanian et al., 2012). In addition, there were increases in multiple transcripts that encode complement factors and chemokine ligands that are expressed by microglia (Zhang et al., 2014) that account for 39% of the elevated transcripts. KEGG pathway analysis indicated that complement, chemokine signaling, and Toll-like receptor pathways are increased with loss of ZFP191 in the whole brain. Unlike the transcripts that had decreased expression, which were mostly associated with oligodendrocyte lineage cells (Figure 1(a)), transcripts that have higher expression levels with loss of ZFP191 were more often noncell specific or associated with gliosis (Figure 1(b)). This may suggest that loss of *Zfp191* expression may perturb cells indirectly by disrupting normal oligodendrocyte maturation and function.

**Table 2.** Top 10 Transcripts That Have Increased Expression in *Zfp191*^*null*^ Whole Brains.

Gene	Fold change	*Zfp191*^*null*^ RPKM
Genes sorted by *Zfp191null* transcript RPKM		
*Apoe*	1.60	1050.00
*Mt1*	1.61	452.00
*Mt2*	1.53	303.00
*Fam107a*	1.70	161.00
*Gfap*	1.97	106.00
*Ctss*	1.69	33.90
*C1qa*	1.66	29.00
*C1qb*	1.70	28.20
*Gabra2*	1.62	21.40
*C1qc*	1.82	16.20

RNA sequencing was performed on *Zfp191*^*null*^ and *Zfp191*^*+/+*^ whole brains (*n* = 2 for each genotype). Transcripts listed for *Zfp191*^*null*^ whole brains had a *p* < .001 fold change when comparing *Zfp191*^*null*^ versus *Zfp191*^*+/+*^ whole brains and are sorted by RPKM value.

### Loss of ZFP191 Perturbs the Transcriptome of OPCs

Because the loss of ZFP191 in the whole brain predominantly disrupts oligodendrocyte lineage-specific transcripts, we wanted to further explore this effect in purified primary cells. We therefore isolated and cultured OPCs from both *Zfp191*^*null*^ and *Zfp191*^*+/+*^ brains. Half of these cells were allowed to differentiate into mature oligodendrocytes. Both cell types from each genotype were then collected for RNA sequencing analysis. We discovered that the absence of ZFP191 during the OPC stage perturbs 444 transcripts (Tables S4 and S5), of which 160 transcripts had decreased expression. Tables 3A and B lists the transcripts that are most abundant in the *Zfp191*^*+/+*^ OPCs and their corresponding reduction with the loss of ZFP191. Transcripts involved in many signal transduction pathways were disturbed including delta-like 1 and 3 (*Dll1, Dll3*), which are involved in the Notch pathway and SRY (sex determining region Y)-box 9 (*Sox9*), which is involved in cAMP signaling (Grandbarbe et al., 2003; Tsuda et al., 2003). KEGG analysis demonstrated that Notch, mitogen-activated protein kinase (MAPK), calcium signaling, and focal adhesion pathways were disrupted with loss of ZFP191 in OPCs.

**Table 3.** Top 10 Transcripts That Have Reduced Expression in *Zfp191*^*null*^ OPCs.

Gene	Fold change	*Zfp191*^*+/+*^ RPKM
A. Genes sorted by *Zfp191*^*+/+*^ transcript RPKM		
*Ptn*	1.77	265.00
*Bcan*	1.99	178.00
*Gpr56*	2.17	119.00
*Add3*	2.18	101.00
*Dll1*	1.82	61.50
*Tubb6*	2.78	63.40
*Rev3l*	1.98	43.90
*Chrna4*	2.33	47.10
*Pnmal2*	1.75	35.30
*Slc38a1*	1.78	34.70
B. Genes sorted by fold change for *Zfp191*^*+/+*^		
*Matn4*	27.70	27.40
*Pstpip2*	15.27	16.70
*Pcdh11x*	5.77	9.14
*9030025P20Rik*	5.24	10.90
*Dll3*	4.92	38.60
*Col18a1*	4.71	10.20
*Hddc3*	4.15	30.10
*Tmem179*	4.13	11.70
*Kifc3*	3.98	59.10
*Lmo3*	3.92	15.30

RNA sequencing was performed on RNA samples isolated from *Zfp191*^*null*^ and *Zfp191*^*+/+*^ OPCs cultured *in vitro* (*n* = 2 for each genotype). Transcripts listed for *Zfp191*^*+/+*^ OPCs in A had a *p* < .001 fold change when comparing *Zfp191*^*+/+*^ versus *Zfp191*^*null*^ OPCs and are sorted by RPKM value. Transcripts listed for *Zfp191*^*+/+*^ OPCs in B had a RPKM value ≥7, a *p* < .001 fold change when comparing *Zfp191*^*+/+*^ versus *Zfp191*^*null*^ OPCs, and are sorted by fold changes.

Loss of ZFP191 results in 284 transcripts being expressed at a higher level than in *Zfp191*^*+/+*^ OPCs (Table 4A and B). Of these, transcripts such as *Id2*, SRY (sex determining region Y)-box 5 (*Sox5*), and *Nkx6.2* are known to be downregulated when OPCs stop proliferating (Wang et al., 2001). In addition, in the absence of ZFP191, histone deactylase 1 (*Hdac1*) is highly expressed along with wingless-type mouse mammary tumor virus integration site family member 7A (*Wnt7a)*, both of which code for proteins that have been shown to be involved in the maintenance of the progenitor state (Ye et al., 2009; Yuen et al., 2014). Loss of ZFP191 also causes several transcripts associated with disruption to the MAPK and Wnt signaling pathways to be more abundant. Overall, these analyses demonstrate that the *Zfp191* mutation disrupts gene expression in the oligodendrocyte lineage more than previously speculated.

**Table 4.** Top 10 Transcripts That Have Increased Expression in *Zfp191*^*null*^ OPCs.

Gene	Fold change	*Zfp191*^*null*^ RPKM
A. Genes sorted by *Zfp191*^*null*^ transcript RPKM		
*Fscn1*	1.82	360.00
*Rps26*	1.91	249.00
*Npy*	64.28	214.00
*Vgf*	24.33	214.00
*Olfm1*	8.11	131.00
*Id2*	3.80	131.00
*Wnk2*	20.09	123.00
*Tgfa*	1.76	116.00
*Elfn1*	3.90	89.80
*Mn1*	50.44	79.80
B. Genes sorted by fold change for *Zfp191*^*null*^		
*Pax7*	24760.00	15.40
*Tmem119*	936.10	7.82
*Epha2*	934.90	41.10
*Rps3a*	261.00	44.90
*Rps18*	240.60	42.10
*Ebi3*	72.15	17.10
*Npy*	64.28	214.00
*Mn1*	50.44	79.80
*Kcnip1*	33.72	73.00
*Tspan18*	31.19	16.60

RNA sequencing was performed on RNA samples isolated from *Zfp191*^*null*^ and *Zfp191*^*+/+*^ OPC cultured *in vitro* (*n* = 2 for each genotype). Transcripts listed for *Zfp191*^*null*^ OPCs in A had a *p* < .001 fold change when comparing *Zfp191*^*null*^ versus *Zfp191*^*+/+*^ OPCs and are sorted by RPKM value. Transcripts listed for *Zfp191*^*null*^ OPCs in B had a RPKM value ≥7, a *p* < .001 fold change when comparing *Zfp191*^*null*^ versus *Zfp191*^*+/+*^ OPCs, and are sorted by fold changes.

### Loss of ZFP191 Perturbs Key Cell Signaling Pathways in Mature Oligodendrocytes

The absence of ZFP191 perturbs the mRNA levels of 5,564 transcripts in mature oligodendrocytes (Tables S6 and S7). Over 45%, or 2,513, transcripts have decreased abundance with loss of ZFP191 in oligodendrocytes. Transcripts for *Sox10, Myrf, Mbp, Mog, Nkx6.2*, and G protein-coupled receptor 37 (*Gpr37)*, all known to code for proteins that play a significant role in myelination (Emery, 2010b), are decreased with loss of *Zfp191* expression (Table 5A and B). Many KEGG-identified pathways are perturbed in mutant oligodendrocytes including regulation of actin cytoskeleton, receptor tyrosine-protein kinase erbB-3 (Erbb), MAPK, sphingomyelin metabolism, and focal adhesion. Transcripts that were more abundant (3,051) in the *Zfp191*^*null*^ oligodendrocytes compared with *Zfp191*^*+/+*^ oligodendrocytes include PDGF receptor alpha polypeptide (*PDGFra)*, sex determining region Y-box 4 (*Sox4*), and myelocytomatosis oncogene (*c-Myc*) along with the known differentiation repressors *Id2/4* and *Hes1/5* (Table 6A and B).

**Table 5.** Top 10 Transcripts That Have Reduced Expression in *Zfp191*^*null*^ Oligodendrocytes.

Gene	Fold change	*Zfp191*^*+/+*^ RPKM
A. Genes sorted by *Zfp191*^*+/+*^ transcript RPKM		
*Plp1*	6.64	8140.00
*Tubb4*	3.53	3790.00
*Cldn11*	5.27	3730.00
*Mbp*	4.82	3560.00
*Mag*	12.04	2670.00
*Cnp*	4.07	2380.00
*Fth1*	2.03	1740.00
*Mal*	13.77	1180.00
*Aplp1*	3.01	852.00
*Mobp*	17.51	841.00
B. Genes sorted by fold change for *Zfp191*^*+/+*^		
*Gjb1*	381.34	181.00
*Ly6a*	132.40	19.30
*Clmn*	102.45	47.10
*Vamp5*	72.49	18.70
*Rab37*	64.52	7.69
*Esyt3*	63.85	7.10
*C030030A07Rik*	60.74	14.90
*Pls1*	57.19	54.70
*Padi2*	56.62	88.90
*Pdlim2*	46.26	180.00

RNA sequencing was performed on RNA samples isolated from *Zfp191*^*null*^ and *Zfp191*^*+/+*^ oligodendrocytes cultured *in vitro* (*n* = 2 for each genotype). Transcripts listed for *Zfp191*^*+/+*^ oligodendrocytes in A had a *p* < .001 fold change when comparing *Zfp191*^*+/+*^ versus *Zfp191*^*null*^ oligodendrocytes and are sorted by RPKM value. Transcripts listed for *Zfp191*^*+/+*^ oligodendrocytes in B had a RPKM value ≥7, a *p* < .001 fold change when comparing *Zfp191*^*+/+*^ versus *Zfp191*^*null*^ oligodendrocytes, and are sorted by fold changes.

**Table 6.** Top 10 Transcripts That Have Increased Expression in *Zfp191*^*null*^ Oligodendrocytes.

Gene	Fold change	*Zfp191*^*null*^ RPKM
A. Genes sorted by *Zfp191*^*null*^ transcript RPKM		
*mt-Nd2*	1.81	1610.00
*Gfap*	14.52	989.00
*Fabp7*	7.48	832.00
*Vim*	15.77	805.00
*Apoe*	5.10	795.00
*Mt3*	21.71	740.00
*Ckb*	2.02	612.00
*Nnat*	7.80	491.00
*Mt1*	2.50	348.00
*Marcks*	3.30	344.00
B. Genes sorted by fold change for *Zfp191*^*null*^		
*Rps18*	2185.00	14.60
*Rps3a*	390.50	16.40
*Npy*	299.90	14.00
*Acta2*	253.00	33.40
*Sod3*	180.50	14.10
*Ccnd2*	166.80	46.50
*Myl9*	139.40	30.30
*Ctgf*	137.20	8.62
*Glycam1*	127.60	26.10
*Gpc4*	116.10	26.20

RNA sequencing was performed on RNA samples isolated from *Zfp191*^*null*^ and *Zfp191*^*+/+*^ oligodendrocytes cultured *in vitro* (*n* = 2 for each genotype). Transcripts listed for *Zfp191*^*null*^ oligodendrocytes in A had a *p* < .001 fold change when comparing *Zfp191*^*null*^ versus *Zfp191*^*+/+*^ oligodendrocytes and are sorted by RPKM value. Transcripts listed for *Zfp191*^*null*^ oligodendrocytes in B had an RPKM value ≥7, a *p* < .001 fold change when comparing *Zfp191*^*null*^ versus *Zfp191*^*+/+*^ oligodendrocytes, and are sorted by fold changes.

**Figure 2.**
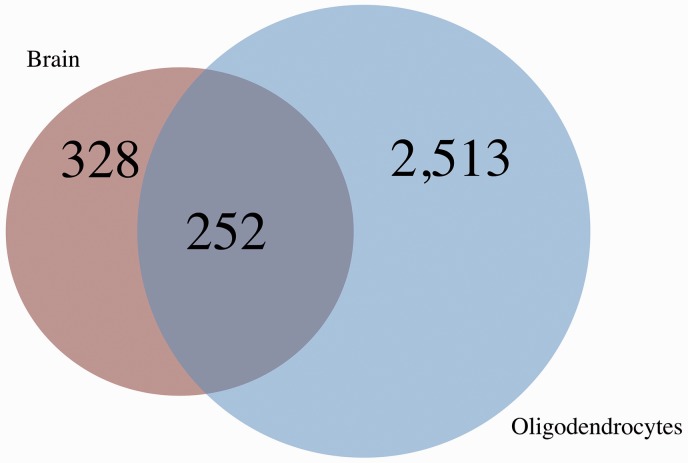
A Venn diagram comparing the number of transcripts with decreased expression in *Zfp191*^*−/−*^ whole brain compared with *Zfp191*^*+/+*^ whole brain (red) and transcripts with decreased expression in *Zfp191*^*−/−*^ oligodendrocytes compared with *Zfp191*^*+/+*^ oligodendrocytes (blue).

Transcripts that are decreased due to loss of ZFP191 in cultured primary mature oligodendrocytes represent nearly 77% of transcripts that are also decreased in the whole brain (327 total transcripts decreased in the whole brain, 252 of which are also decreased in cultured primary mature oligodendrocytes; Figure 2). The transcripts that are decreased in both datasets include critical myelin-associated transcripts such as 2′,3′-cyclic nucleotide 3′ phosphodiesterase (*Cnp*), aspartoacylase (*Aspa*), myelin and lymphocyte protein T cell differentiation protein (*Mal1*), proteolipid protein 1 (*Plp1*), myelin-associated oligodendrocyte basic protein (*Mobp*), *Mag*, and *Mbp* along with the key transcription factors *Sox10, Myrf*, and *Nkx6-2* (Emery, 2010a, 2010b). In addition, transcripts for key factors regulating transcription such as *Gpr37*, mothers against decapentaplegic homolog 7 (*Smad7*), receptor tyrosine-protein kinase erbB-3, survival of motor neuron protein-interacting protein 1 (*Sip1/Zeb2*; Brinkmann et al., 2008; Weng et al., 2012) along with transcripts for enzymes that play a key role in lipid biogenesis, such as elongation of very long chain fatty acids protein 7 (*Elovl7*) and ceramide synthase 2 (*Lass2*), are decreased with loss of ZFP191. In contrast, very few (14) genes have increased mRNA levels with loss of ZFP191 in both the whole brain and mature oligodendrocytes; these include inhibitor of differentiation 3 (*Id3*), lunatic fringe (*Lfng*), and apolipoprotein E (*Apoe*).

**Figure 3.**
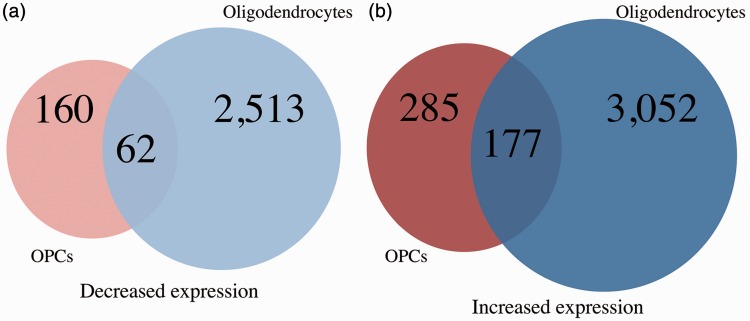
A Venn diagram comparing the number of transcripts (A) with decreased expression in *Zfp191*^*−/−*^ OPCs compared with *Zfp191*^*+/+*^ OPCs (light red) and *Zfp191*^*−/−*^ oligodendrocytes compared with *Zfp191*^*+/+*^ oligodendrocytes (light blue). A Venn diagram comparing transcripts (B) with increased expression in *Zfp191*^−*/*−^ OPCs compared with *Zfp191*^*+/+*^ OPCs (dark red) and *Zfp191*^−*/*−^ oligodendrocytes compared with *Zfp191*^*+/+*^ oligodendrocytes (dark blue). *Note*. OPCs = oligodendrocyte progenitor cell.

There are 177 transcripts that have increased levels in both OPCs and oligodendrocytes that do not express ZFP191 (Figure 3). Several of these transcripts encode proteins that play a role in the control of oligodendrocyte development by repressing maturation such as *Id2, Wnt5a/7a, Hdac1, Sox5*, and paired-box 7 (*Pax7*; Liu et al., 2006; Emery, 2010a, 2010b; Fulton et al., 2011; Kuspert and Wegner, 2015). We noted that 1,522 transcripts that are normally decreased upon differentiation from the OPC to oligodendrocyte stage (Zhang et al., 2014) are not suppressed in mature oligodendrocytes that lack ZFP191. These transcripts include *Dll1, Ascl1/Mash1, c-Myc, Notch1, Pdgfra*, and *Sox2/4/6/9/21*. Many of these factors are known to play a role in transcriptional regulation. Elevation of these transcript levels may inappropriately maintain the expression of downstream genes that promote OPC identity and prevent maturation.

### Transcription Factor Expression Is Perturbed With Loss of *Zfp191*

Loss of *Zfp191* expression in the brain perturbs the expression of 25 genes that encode transcription factors as identified by the Animal Transcription Factor Database (AnimalTFDB) and Riken Transcription Factor Database (TFdb; (Kanamori et al., 2004; Zhang et al., 2012). A majority of these transcripts (21) have reduced expression levels in *Zfp191*^*null*^ brain.

In isolated and cultured OPCs, loss of *Zfp191* expression perturbs the expression of 58 transcription factor transcripts. In contrast to the whole brain data, the majority of these transcripts have increased expression (41) with loss of ZFP191. *Pax7* has an approximately 24,000 fold increase in *ZFP191*^*null*^ OPCs (this high fold change is due to the absence of detectable *Pax7* mRNA in *Zfp191*^*+/+*^ cells). In cultured oligodendrocytes, we identified 558 transcripts that encode putative transcription factors that have perturbed expression with the loss of ZFP191. Table 7 lists the 10 transcripts that have the greatest fold change in *Zfp191*^*null*^ oligodendrocytes. Our data demonstrate that loss of ZFP191 reduces expression of *Myrf, Nkx6-2*, and *Olig1*, all of which play a vital role in oligodendrocyte differentiation and myelination (Emery, 2010a, 2010b). As with the OPCs, loss of ZFP191 in oligodendrocytes results in more increases in transcription factor transcripts (333) than decreases. Similar to what was seen with OPCs, *Pax7* has the largest fold change (241-fold) in *Zfp191*^*null*^ oligodendrocytes.

**Table 7.** Top 10 Transcription Factor Transcripts With Reduced Expression in *Zfp191*^*null*^ Cultured Oligodendrocytes.

Gene	Fold change	*Zfp191*^*+/+*^ RPKM
Transcription factors with reduced expression in *Zfp191*^*null*^ cultured oligodendrocytes		
*Scx*	26.29	8.14
*Onecut2*	20.52	4.06
Zfp457*	18.23	1.11
*Irx5*	15.74	1.98
*Pou3f1*	15.52	19.3
*Utf1*	14.07	1.17
*Mafa*	11.69	1.07
Tle6*	10.19	3.43
Hes3*	8.96	11.3
Rbpjl*	8.40	2.23

RNA sequencing was performed on RNA samples isolated from *Zfp191*^*null*^ and *Zfp191*^*+/+*^ oligodendrocytes cultured *in vitro* (*n* = 2 for each genotype). Transcription factor transcripts were compiled from AnimalTFDB and TFdb (Kanamori et al., 2004; Zhang et al., 2012). Transcription factor transcripts listed for *Zfp191*^*+/+*^ whole brains had a RPKM value ≥0.5, a ≥ 1.5-fold change when comparing *Zfp191*^*+/+*^ versus *Zfp191*^*null*^ oligodendrocytes, *p* < .001, and sorted by RPKM value. *=Gene expression is also reduced in *Zfp191*^*null*^ OPCs.

### The Perturbation of the Transcriptome Is Primarily Due to Loss of ZFP191 Rather Than the Absence of Myelin

The lack of ZFP191 results in profound CNS hypomyelination, which might have a secondary effect on the transcriptome. To distinguish transcripts that are affected primarily through the loss of ZFP191 from those that are perturbed due to the loss of myelin, we examined the transcriptome of the well-characterized hypomyelinated *shiverer* mouse, in which the hypomyelination is the result of a spontaneous null mutation in the *Mbp* gene (Readhead et al., 1987). We performed RNA sequencing on P21 *shiverer* whole-brain RNA samples, as well as RNA isolated from primary OPCs and oligodendrocytes similar to the *Zfp191*^*−/−*^ datasets. The loss of *Mbp* in P21 whole brain results in the perturbation of 93 transcripts (Tables S8 and S9). The majority of these transcripts were expressed at a higher level in *shiverer* mutant mice (67 transcripts; Tables 8A and B, 9A and B). Transcripts that were more abundant in the *shiverer* brain include *Nkx6-2, Erbb3* and *Apoe*. Transcripts that had a lower expression level in the *shiverer* mouse brains compared with *Mbp*^*+/+*^ littermates include *Mbp*, UDP galactosyltransferase 8A (*Ugt8a*), *Fa2h, Mal, gelsolin* (*Gsn*), lanosterol synthase (*Lss*), squalene epoxidase (*Sqle*), *Hmgcr*, and *Plp1*. Examining the altered transcripts for KEGG pathway analysis revealed that the loss of MBP in the whole brain perturbs phosphatidylinositol signaling along with sterol biosynthesis.

**Table 8.** Top 10 Transcripts That Have Decreased Expression in *Shiverer* Whole Brains.

Gene	Fold change	*Mbp*^*+/+*^ RPKM
A. Genes sorted by *Mbp*^*+/+*^ transcript RPKM		
*Plp1*	1.61	1120.00
*Mbp*	33.68	506.00
*Mal*	2.23	178.00
*Ugt8a*	3.19	122.00
*Trf*	2.31	69.30
*Scd1*	1.83	44.00
*Apod*	2.17	40.30
*Gsn*	1.81	33.60
*Hmgcr*	1.61	29.90
*Fa2h*	2.74	26.40
B. Genes sorted by fold change for *Mbp*^*+/+*^ transcripts ≥ 7 RPKM		
*Mbp*	33.68	506.00
*Ugt8a*	3.19	122.00
*Fa2h*	2.74	26.40
*Trf*	2.31	69.30
*Mal*	2.23	178.00
*Apod*	2.17	40.30
*Anln*	2.113	8.02
*Sc4mol*	2.012	14.1

RNA sequencing was performed on *Mbp*^*shi*^ and *Mbp*^*+/+*^ whole-brain RNA samples (*n* = 2 for each genotype). Transcripts listed for *Mbp*^*+/+*^ whole brains in A had a *p* < .001 fold change when comparing *Mbp*^*+/+*^ versus *Mbp*^*shi*^ whole brains and are sorted by RPKM value. Transcripts listed for *Mbp*^*+/+*^ whole brains in B had an RPKM value ≥7, a *p* < .001 fold change when comparing *Mbp*^*+/+*^ versus *Mbp*^*shi*^ whole brains, and are sorted by fold changes.

**Table 9.** Top 10 Transcripts That Have Increased Expression in *Shiverer* Whole Brains.

Gene	Fold change	*Mbp*^*shi*^ RPKM
A. Genes sorted by *Mbp*^*shi*^ transcript RPKM		
*Apoe*	1.60	752.00
*Ptms*	1.62	228.00
*Ptgds*	1.98	138.00
*Csrp1*	1.72	87.80
*Sirt2*	1.84	70.90
*S100a1*	1.76	55.80
*Nfasc*	1.71	53.90
*Map4k4*	1.53	45.60
*P4hb*	1.52	41.00
*Nudc*	1.53	38.20
B. Genes sorted by fold change for *Mbp*^*shi*^ transcripts ≥ 7 RPKM		
*Gsbs*	3.39	28.10
*Car14*	2.77	14.80
*Pls1*	2.70	7.77
*Tmem2*	2.15	8.09
*Phlda1*	2.05	14.10
*H2afj*	2.04	17.60
*Adamts4*	2.04	15.00
*Rras2*	2.02	17.60
*Ptgds*	1.98	138.00
*Hsd17b7*	1.98	10.10

RNA sequencing was performed on *Mbp*^*shi*^ and *Mbp*^*+/+*^ whole-brain RNA samples (*n* = 2 for each genotype). Transcripts listed for *Mbp*^*shi*^ whole brains in A had a *p* < 0.001 fold change when comparing *Mbp*^*shi*^ versus *Mbp*^*+/+*^ whole brains and are sorted by RPKM value. Transcripts listed for *Mbp*^*shi*^ whole brains in B had an RPKM value ≥7, a *p* < .001 fold change when comparing *Mbp*^*shi*^ versus *Mbp*^*+/+*^ whole brains, and are sorted by fold changes.

*Mbp* expression loss in OPCs resulted in the perturbation of 10 transcripts, all of which had low RPKM values and small changes (Tables S10 and S11). This is expected as *Mbp* is upregulated upon exit of the OPC stage. The loss of MBP in mature oligodendrocytes perturbs 387 transcripts; 311 of those transcripts are expressed at a lower abundance with the absence of MBP (Tables S12 and S13; Table 10A and B along with Table 11A and B).

**Table 10.** Top 10 Transcripts That Have Reduced Expression in *Shiverer* Oligodendrocytes.

Gene	Fold change	*Mbp*^*+/+*^ RPKM
A. Genes sorted by *Mbp*^*+/+*^ transcript RPKM		
*Mbp*	99.90	3150.00
*Ugt8a*	1.52	721.00
*Ddr1*	1.52	517.00
*Mog*	1.78	498.00
*Phyhipl*	1.54	417.00
*Glul*	1.56	415.00
*Ptgds*	2.92	377.00
*Nrbp2*	1.56	268.00
*Cryab*	2.72	252.00
*Qdpr*	1.70	239.00
B. Genes sorted by fold change for *Mbp*^*+/+*^ transcripts ≥ 7 RPKM		
*Mbp*	99.90	3150.00
*Stac2*	5.75	7.18
*Aqp1*	3.95	9.78
*Hrk*	3.94	8.30
*Eef1a2*	3.54	7.88
*Ly6a*	3.36	17.10
*Atp1a3*	3.14	10.90
*Ptgds*	2.92	377.00
*A830039N20Rik*	2.85	12.70
*Kcnma1*	2.85	7.28

RNA sequencing was performed on RNA isolated from *Mbp*^*shi*^ and *Mbp*^*+/+*^ oligodendrocytes cultured *in vitro* (*n* = 2 for each genotype). Transcripts listed for *Mbp*^*+/+*^ oligodendrocytes in A had a *p* < .001 fold change when comparing *Mbp*^*+/+*^ versus *Mbp*^*shi*^ in oligodendrocytes and are sorted by RPKM value. Transcripts listed for *Mbp*^*+/+*^ oligodendrocytes in B had a RPKM value ≥7, a *p* < .001 fold change when comparing *Mbp*^*+/+*^ versus *Mbp*^*shi*^ in oligodendrocytes, and are sorted by fold changes.

**Table 11.** Top 10 Transcripts That Have Increased Expression in Shiverer Oligodendrocytes.

Gene	Fold change	*Mbp*^*shi*^ RPKM
A. Genes sorted by *Mbp*^*shi*^ transcript RPKM		
*Cyb5*	1.72	153.00
*Rap2a*	1.76	85.80
*Chst2*	1.53	84.40
*Sema6a*	1.59	82.20
*Sema6d*	1.68	60.40
*Mmd2*	1.67	54.80
*Dusp15*	1.58	49.50
*Cntn1*	1.56	45.70
*Mapre2*	1.71	39.30
*Fam107b*	2.17	34.50
B. Genes sorted by fold change for *Mbp*^*shi*^ transcripts > 7 RPKM		
*Dct*	6.32	16.50
*Alox5*	2.80	14.60
*Fam107b*	2.17	34.50
*Daam2*	1.93	9.02
*Tmprss5*	1.83	11.20
*Capn5*	1.80	24.70
*Rap2a*	1.76	85.80
*Ndrg2*	1.75	17.80
*Kcna2*	1.74	20.00
*Id4*	1.72	8.26

RNA sequencing was performed on RNA samples isolated from *Mbp*^*shi*^ and *Mbp*^*+/+*^ oligodendrocytes cultured *in vitro* (*n* = 2 for each genotype). Transcripts listed for *Mbp*^*shi*^ oligodendrocytes in A had a *p* < .001 fold change when comparing *Mbp*^*shi*^ versus *Mbp*^*+/+*^ in oligodendrocytes and are sorted by RPKM value. Transcripts listed for *Mbp*^*shi*^ oligodendrocytes in B had an RPKM value ≥7, a *p* < .001 fold change when comparing *Mbp*^*shi*^ versus *Mbp*^*+/+*^ in oligodendrocytes, and are sorted by fold changes.

**Figure 4.**
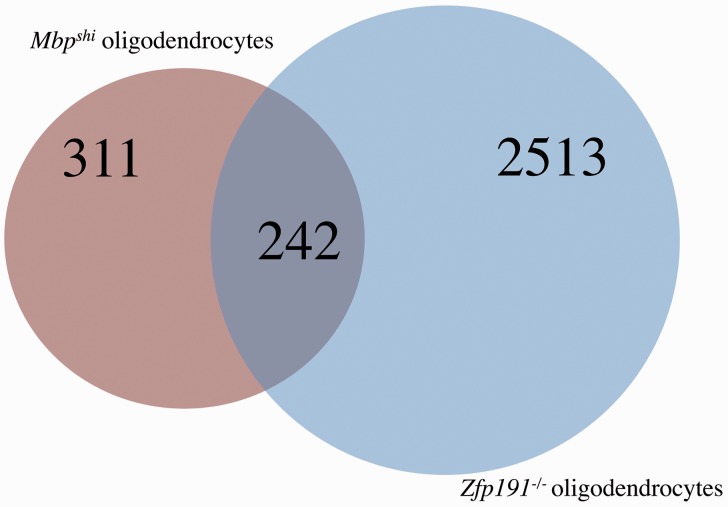
A Venn diagram comparing transcripts with decreased expression in *Mbp^shi^ oligodendrocytes* compared with *Mbp*^*+/+*^ oligodendrocytes (red) and transcripts with decreased expression in *Zfp191*^−*/*−^ oligodendrocytes compared with *Zfp191*^*+/+*^ oligodendrocytes (blue).

**Figure 5.**
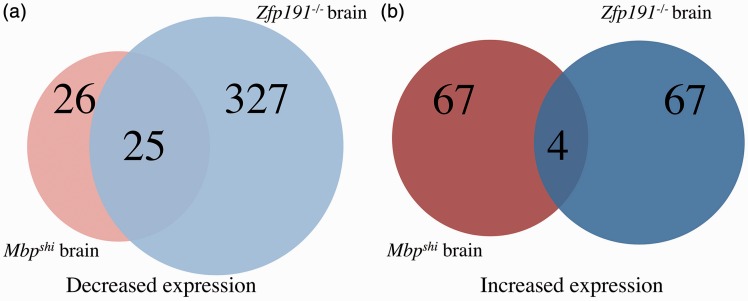
A Venn diagram comparing transcripts (A) with decreased expression in *Mbp*^*shi*^ whole brain compared with *Mbp*^*+/+*^ whole brain (red) and *Zfp191*^−*/*−^ whole brain compared with *Zfp191*^*+/+*^ whole brain (blue). A Venn diagram comparing transcripts (B) with increased expression in *Mbp*^*shi*^ whole brain compared with *Mbp*^*+/+*^ whole brain (red) and *Zfp191*^−*/*−^ whole brain compared with *Zfp191*^*+/+*^ whole brain (blue).

It is striking that approximately 75% of the transcripts that are reduced with loss of *Mbp* expression are also reduced with the loss of *Zfp191* expression (Figure 4). Transcripts that have reduced expression include *Mbp, Mog, Ugt8a*, and *Fasn*, all key transcripts involved in oligodendrocyte development and maturation. When assessing the transcriptional differences detected in total brain RNA, the comparison is even more striking as 25 of the 26 transcripts that have decreased expression with loss of *Mbp* expression are also decreased with loss of *Zfp191*^−/−^ expression (Figure 5(a)). Nevertheless, there are only four transcripts that have increased expression in both mouse models of hypomyelination in the whole brain (Figure 5(b)). It is clear from these analyses that the vast majority of genes that demonstrate altered expression in the *Zfp191* mutants are expressed normally in *Mbp*^*shi*^ mice.

### Disrupted Cholesterol Biosynthesis Is a Transcriptional Fingerprint of Hypomyelination

**Figure 6.**
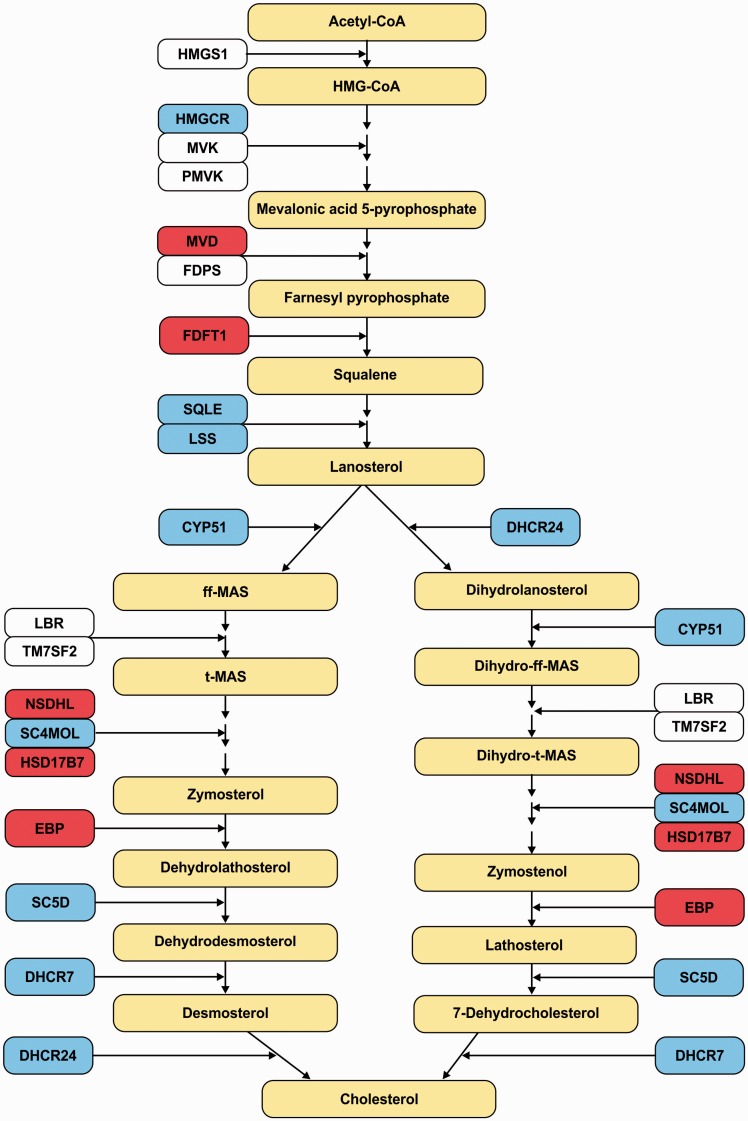
Cholesterol biosynthesis pathway transcripts are disrupted in hypomyelinated brains. Yellow boxes denote chemical compounds involved in this pathway. White, blue, or red boxes denote the transcripts involved in this pathway. Red boxes denote transcripts that have reduced expression in the *Zfp191*^*null*^ whole brain. Blue boxes denote transcripts that have reduced expression in both the *Zfp191*^*null*^ and *shiverer* whole brains. White boxes denote transcripts that are not affected.

The cholesterol biosynthetic pathway is disrupted in both the *Zfp191* and *Mbp* mutants examined here. Table 12 shows that both mouse models have reduced expression of a number of transcripts required for proper cholesterol biosynthesis, including the rate-limiting enzyme HMGCR. The disruption of the cholesterol biosynthesis pathway in these two models suggests that this is a transcriptional signature of hypomyelination (Figure 6). Interestingly, the expression level of these transcripts was not perturbed in isolated *Zfp191*^*null*^ or *shiverer* OPCs or oligodendrocytes. Unlike the oligodendrocytes in the brain, primary cultured oligodendrocytes do not make compact myelin. Therefore, we propose that the observed perturbation in the cholesterol biosynthetic pathway observed only in the brain and not in the primary cultured cells is secondary to the lack of myelin production.

**Table 12.** Genes Perturbed in Fatty Acid Degradation for Cholesterol Production.

	*Zfp191*^−/−^ brain		*Shiverer* brain	
Gene	Fold change	RPKM for *Zfp191*^*+/+*^	Fold change	RPKM for *Mbp*^*+/+*^
*Lss*	2.13	7.50	1.72	6.62
*Dhcr7*	1.71	9.78	1.60	8.53
*Sc5d*	1.82	29.50	1.59	19.30
*Sqle*	1.82	26.50	1.68	24.90
*Cyp51*	1.99	23.20	1.93	20.90
*Sc4mol*	2.21	16.80	2.01	14.10
*Hmgcr*	1.66	28.20	1.60	29.90
*Fdft1*	1.80	19.00	1.61	14.30
*Mvd*	1.76	9.34	N.S.	7.47
*Nsdhl*	1.70	7.97	N.S.	9.17

RNA sequencing was performed on *Zfp191*^*+/+*^ and *Zfp191*^*null*^ whole-brain RNA samples along with *Mbp*^*+/+*^ and *Mbp*^*shi*^ whole-brain RNA samples (*n* = 2 for each genotype). Transcripts listed had a RPKM value ≥0.5, a ≥ 1.5-fold change when comparing *Zfp191*^*+/+*^ versus *Zfp191*^*null*^ and *Mbp*^*+/+*^ versus *Mbp*^*shi*^ in whole brains, and *p* < .001. N.S. = not significant.

To confirm that the expression levels of key cholesterol biosynthetic transcripts are perturbed, reverse-transcriptase PCR (RT-PCR) was performed (Figure S1).

## Discussion

### ZFP191 Plays a Key Role in Oligodendrocyte Development and Myelination

In the current study, we examined the role that the putative transcription factor ZFP191 has on the integrity of the transcriptome using high-throughput sequencing of RNA isolated from total brain, as well as isolated oligodendrocyte lineage cells. We first examined how loss of ZFP191 affects the transcriptome of the P21 whole mouse brain. We find that most transcripts that had decreased expression with loss of *Zfp191* are associated with oligodendrocyte lineage cells, which is consistent with the hypomyelinating phenotype that these mutant animals display (Howng et al., 2010). Transcripts that have increased expression as a result of loss of *Zfp191* expression are associated with gliosis. This gliotic response may be the result of loss of *Zfp191* expression in astrocytes and microglia, but it could also reflect a secondary response to the impairment of oligodendrocyte function and myelin production. Loss of *Zfp191* expression perturbs the transcriptome of *in vitro* cultured OPCs. This implies that ZFP191 has a function prior to the generation of oligodendrocytes. The loss of *Zfp191* expression in isolated oligodendrocytes results in a perturbation of ∼5,500 transcripts. Transcripts, such as *Mbp, Mog, Mobp*, and *Cnp*, for proteins involved in the generation and function of myelin were decreased with loss of *Zfp191* expression. The majority of transcripts (∼75%) that had lower expression in the *Zfp191*^−*/−*^ whole brain also had lower expression in *Zfp191*^*−/−*^ oligodendrocytes (Figure 2), demonstrating that the perturbation of the whole brain transcriptome is due mainly to the perturbation of oligodendrocytes.

Recently, it has been reported that patients hemizygous for 18q chromosomal deletions that include the region that contains the *Zfp191* human ortholog *ZNF24* display seizures and tremors, suggestive of myelin abnormalities (Cody et al., 2015). Interestingly, OPCs and oligodendrocytes that are heterozygous for the *Zfp191* null mutation display transcriptome alterations similar to the homozygous mutant cells, albeit to a lesser extent (data not shown). This raises the possibility that a subset of human disorders with 18q deletions might be due, at least in part, to *ZNF24* haploinsufficiency.

### Zfp191 Plays a Role in the Transcriptional Networks That Govern Oligodendrocyte Differentiation

Due to the large number of transcripts perturbed from lack of *Zfp191* expression, we examined whether transcription factor networks were disrupted. Indeed, with loss of *Zfp191* expression, several critical transcription factor transcripts such as *Nkx6.2, Sox10*, and *Myrf* had decreased expression in cultured oligodendrocytes (Hornig et al., 2013). These transcription factors are mandatory for oligodendrocyte differentiation, as shown in genetic ablation studies (Stolt et al., 2002; Koenning et al., 2012; Mei et al., 2013). These data raise the possibility that ZFP191 functions, at least in part, upstream of many critical oligodendrocyte transcription factors including *Sox10* and *Myrf*.

In the absence of *Zfp191* expression, the transcripts for transcriptional repressors *Id2/4* and *Hes1/5* are increased in *Zfp191*^*null*^ oligodendrocytes. Normally, the expression of these genes decreases with differentiation from the OPC to mature oligodendrocyte stage (Dugas et al., 2006; Zhang et al., 2014), and this downregulation has been shown to be required for the proper differentiation of OPCs into mature oligodendrocytes (Norton, 2000; Wang et al., 2001; Wu et al., 2003; Liu et al., 2006; Ogata et al., 2011). The continued expression of these transcripts suggests that *Zfp191*^*null*^ oligodendrocytes have not fully differentiated into cells capable of myelinating axons. ZFP191 may play a direct role in downregulating these transcripts or this may represent a secondary effect associated with the absence of ZFP191.

### ZFP191 Plays a Role in Signaling Cascades That Control Oligodendrocyte Differentiation

The Notch signaling pathway has been shown to be important for oligodendrocyte differentiation (Hu et al., 2003; Popko, 2003). We find that the transcripts of the Notch signaling effectors *Dll3* and *Lfng* along with the downstream target recombination signal binding protein for immunoglobulin kappa J region-like *(Rbpjl)* are reduced in OPCs derived from the *Zfp191*^*null*^ mice. These data suggest that ZFP191 may play a role in the regulation of the Notch pathway in oligodendrocytes and through its absence may prevent proper maturation and myelin production. Our data show that several additional pathways are perturbed with loss of *Zfp191* expression such as the Erbb, MAPK, and Wnt signaling cascades.

The inhibitory effect of the Wnt signaling pathway is critical for oligodendrocyte differentiation (Fancy et al., 2009; Rosenberg and Chan, 2009). We find that the Wnt signaling effectors low-density lipoprotein receptor-related protein 5, frizzled homolog 1, and smoothened homolog 1 (*Lrp5, Fzd1*, and *Smo1*) are increased in the oligodendrocytes derived from the *Zfp191*^*null*^ mice. These data suggest that ZFP191 may play a role in proper regulation of the Wnt pathway in oligodendrocytes.

In addition, loss of *Zfp191* affects the expression of *Wnt7a*, which is detected at approximately 20-fold higher level in the mutant oligodendrocytes. WNT7A has been shown to affect the developing brain as both an autocrine and a paracrine signaling molecule (Qu et al., 2013). Functioning as an autocrine signaling molecule, WNT7a maintains OPCs in their progenitor state and prevents differentiation. When functioning as a paracrine signaling molecule, WNT7a promotes endothelial cells to initiate angiogenesis in white matter areas (Yuen et al., 2014). The perturbed expression of *Wnt7a* in the *Zfp191*^*null*^-derived oligodendrocyte lineage cells may suggest that ablation of *Zfp191* may affect other cell types in the brain in a paracrine fashion mediated by WNT7A. This may explain some of the differences detected between the whole brain transcriptome compared with isolated oligodendrocyte lineage cells and may explain, at least in part, the effect of ablation of *Zfp191* on non-oligodendrocyte lineage cell-specific transcripts in the brain.

### The Disruption of the Oligodendrocyte Transcriptome Primarily Results From the Loss of ZFP191 Not the Absence of Myelin Production

To examine whether the altered gene expression in the *Zfp191* mutant CNS is due to the direct loss of ZFP191 or whether it represents a secondary consequence of the inability to produce myelin, we performed RNA sequencing using the *shiverer* mouse. The loss of MBP results in 75% fewer transcripts being perturbed in the whole brain than in the *Zfp191*^*null*^ mouse. We also observed that fewer transcripts associated with transcriptional control were perturbed with loss of *Mbp* expression. This seems reasonable as MBP is a structural protein, but it is important to note that expression of *Id2/4, Runx1, Atf3*, and *Scx* are altered in both *Zfp191*^*null*^ and *shiverer* oligodendrocytes suggesting that there may be transcription factors that are affected due to the general loss of proper myelin production. Interestingly, previous studies have shown that a small portion of MBP is localized to the nucleus of oligodendrocytes, which may indicate a possible role for this protein in transcriptional control (Pedraza et al., 1997; Smith et al., 2012; Smith et al., 2013). Nevertheless, a direct regulatory effect of MBP on oligodendrocyte gene expression has not been demonstrated. A majority of the transcripts that are perturbed with loss of *Mbp* expression are also perturbed with loss of *Zfp191* expression in oligodendrocytes (Figure 4). This may demonstrate that ZFP191 plays a key role upstream of *MBP*, particularly as many more transcripts are perturbed with loss of ZFP191 than with MBP.

### Hypomyelination Causes a Reduction in Key Transcripts Involved in the Lipid Biosynthesis Pathway

The absence of myelin in the *Zfp191*^*null*^ and *shiverer* mouse whole brains results in the reduction of a number of transcripts that encode key proteins in the cholesterol and lipid biosynthetic pathways. The loss of either ZFP191 or MBP results in similar reductions of *Hmgcr*, which encodes for the rate-limiting enzyme in cholesterol biosynthesis, and several transcripts that encode for proteins involved in the final steps of this pathway (Figure 6). These data suggest that oligodendrocytes have a feedback system that allows them to regulate myelin lipid synthesis depending on their myelinating state. The control of cholesterol homeostasis is critical for myelination (Saher et al., 2005; Verheijen et al., 2009), and defects in lipid synthesis have been linked to demyelination (Rolyan et al., 2015). The transcription factors sterol regulatory element-binding proteins (SREBPs; encoded by the *SREBF1* and *SREBF2* genes) regulates nine of the genes that encode enzymes required for cholesterol biosynthesis (Sakakura et al., 2001). In *Zfp191*^*null*^ OPCs and oligodendrocytes, *SREBF1* and *SREBF2* are expressed more abundantly than in *Zfp191*^*+/+*^ cells. Although both of the SREBPs transcripts are more abundant, the mRNA levels of their downstream targets such as *Hmgcr, Sqle*, and *Lss* are not altered in *Zfp191*^*null*^ OPCs and oligodendrocytes, suggesting a more complex level of regulation. Moreover, *SREBF1* and *SREBF2* expression is not perturbed in the hypomyelinated *shiverer* mouse, further suggesting that these transcription factors are not the myelin sensors that regulate lipid biosynthesis. Data from these models of hypomyelination may help identify novel potential sensors in oligodendrocytes for proper cholesterol and lipid biosynthesis. Lipid biosynthesis transcripts are also expressed abundantly in many cells in the CNS, particularly astrocytes, which play a key role in providing lipids and cholesterol for neurons (for review, see Pfrieger and Ungerer, 2011). The decrease of key lipid biosynthesis transcripts in these models of hypomyelination may be due to a direct effect on oligodendrocytes or it may reflect a secondary effect of oligodendrocyte perturbation on the homeostatic production of cholesterol by the CNS.

## Conclusion

ZFP191 is a putative transcription factor that plays a critical role in CNS myelination (Emery, 2010a, 2010b; Howng et al., 2010; Yu et al., 2013; Kuspert and Wegner, 2015). Therefore, a complete understanding of ZFP191 function is critical. Our data suggest that ZFP191 plays a key role in the transcriptional network that controls oligodendrocyte development and myelination. Loss of ZFP191 primarily perturbs the mature oligodendrocyte transcriptome, although OPC transcriptome alterations demonstrate that loss of ZFP191 is acting early in the oligodendrocyte lineage. We also show that ZFP191 acts, at least in part, upstream of *Sox10* and *Myrf* and may have a critical role in their transcriptional regulation. Many pathways such as MAPK, Notch, Wnt, and Erbb signaling cascades, which are known to play key roles in oligodendrocyte development, are perturbed with the loss of ZFP191. These data suggest that the loss of ZFP191 results in the subsequent decrease in abundance of key transcription factors along with transcripts that play a key role in signal transduction and cholesterol biosynthesis. Therefore, ZFP191 is a central transcriptional regulator of oligodendrocyte development and myelination.

## Supplementary Material

Supplementary material

## Supplementary Material

Supplementary material

## Supplementary Material

Supplementary material

## Supplementary Material

Supplementary material

## Supplementary Material

Supplementary material

## Supplementary Material

Supplementary material

## Supplementary Material

Supplementary material

## Supplementary Material

Supplementary material

## Supplementary Material

Supplementary material

## Supplementary Material

Supplementary material

## Supplementary Material

Supplementary material

## Supplementary Material

Supplementary material

## Supplementary Material

Supplementary material

## Summary Statement

The transcriptional program of oligodendrocyte maturation and myelination has not been fully characterized. In this study, we use RNA sequencing to analyze how the loss of the transcription factor zinc finger protein 191 results in the perturbation of oligodendrocyte development.

## Supplemental Material

The online figures and tables are available at http://asn.sagepub.com/supplemental.
